# Regulatory Mechanisms of IL-33-ST2-Mediated Allergic Inflammation

**DOI:** 10.3389/fimmu.2018.02004

**Published:** 2018-09-04

**Authors:** Hiroaki Takatori, Sohei Makita, Takashi Ito, Ayako Matsuki, Hiroshi Nakajima

**Affiliations:** ^1^Department of Allergy and Clinical Immunology, Graduate School of Medicine, Chiba University, Chiba, Japan; ^2^Department of Rheumatology, Hamamatsu Medical Center, Hamamatsu, Japan

**Keywords:** IL-33, ST2, innate lymphoid cells (ILCs), regulatory T cells (Tregs), epithelial cells

## Abstract

Interleukin-33 (IL-33) plays multiple roles in tissue homeostasis, prevention of parasitic infection, and induction of allergic inflammation. Especially, IL-33-ST2 (IL-1RL1) axis has been regarded as the villain in allergic diseases such as asthma and atopic dermatitis and in autoimmune diseases such as rheumatoid arthritis. Indeed, a number of studies have indicated that IL-33 produced by endothelial cells and epithelial cells plays a critical role in the activation and expansion of group 2 innate lymphoid cells (ILC2s) which cause allergic inflammation by producing large amounts of IL-5 and IL-13. However, mechanisms that antagonize IL-33-ST2-mediated allergic responses remain largely unknown. Recently, several groups including our group have demonstrated cellular and molecular mechanisms that could suppress excessive activation of ILC2s by the IL-33-ST2 axis. In this review, we summarize recent progress in the regulatory mechanisms of IL-33-ST2-mediated allergic responses. Selective targeting of the IL-33-ST2 axis would be a promising strategy in the treatment of allergic diseases.

## Introduction

Interleukin-33 (IL-33) is a member of the IL-1 cytokine family and mainly expressed in non-hematopoietic cells such as endothelial cells, epithelial cells, fibroblast-like cells, and myofibroblasts during homeostasis and in response to inflammation (1–5). IL-33 functions on target cells by binding to a heterodimeric receptor composed of suppression of tumorigenicity 2 (ST2, also known as IL-1RL1) and co-receptor, IL-1 receptor accessory protein (IL-1RAcP) (6, 7). Triggering of ST2/IL-1RAcP by IL-33 activates intracellular signaling pathways, which is initiated by homotypic protein-protein interactions with the adaptor molecule MyD88 and the further recruitment of IRAKs and TRAF6, leading to the expression of several inflammatory mediators through the activation of nuclear factor-κB (NF-κB) and mitogen-activated protein kinase (MAPK) pathways (8, 9). IL-33 activates a variety of tissue-resident immune cells that express ST2, including group 2 innate lymphoid cells (ILC2s), mast cells, Th2 cells, regulatory T cells (Tregs), natural killer (NK) cells, eosinophils, basophils, dendritic cells, and alternatively activated macrophages (10–12).

Regarding *in vivo* relevance of IL-33-ST2 axis, it has been shown that bronchial epithelium is an important reservoir of IL-33 in the lung and that IL-33 expression is elevated in the airways of bronchial asthma along with disease severity (13). It has also been shown that increased IL-33 expression is associated with enhanced reticular basement membrane thickness in endobronchial tissues in children with severe therapy-resistant asthma (14). In addition, it has been reported that airway remodeling is absent in ST2^−/−^ mice in house dust mite (HDM)-induced neonatal asthma models (14). Moreover, IL-33 expression is not affected by steroid treatment in neonatal HDM-treated mice and in endobronchial tissues from children with severe therapy-resistant asthma (14). These findings suggest that IL-33-ST2 axis causes refractoriness in the allergic asthma.

Recently, numerous studies analyzing patient samples and murine models have revealed that ILC2s produce large amounts of IL-5 and IL-13 in response to IL-33 and IL-25 and that the activation of ILC2s by IL-33-ST2-mediated signaling contributes to anti-helminth responses and to the development of various allergic diseases such as asthma, atopic dermatitis, allergic rhinitis, and chronic rhinosinusitis (10–12, 15, 16). Furthermore, IL-33-ST2-mediated signaling seems to be involved in the development and exacerbation of non-allergic inflammatory conditions including arthritis, chronic obstructive pulmonary disease (COPD) with cigarette smoke, experimental autoimmune encephalomyelitis (EAE), periodontitis, and retina inflammation in murine models (12), although it remains unclear whether IL-33-ST2-ILC2s axis is involved in the pathogenesis of corresponding diseases in humans. Because IL-33-ST2-mediated signaling seems critical for the induction of excessive inflammatory responses in many conditions, it would be worthy of further investigation elucidating regulatory mechanisms against IL-33-ST2-mediated signaling to develop therapeutic strategies for these inflammatory diseases. In this review, we summarize recent progress in the regulatory mechanisms for IL-33-ST2-mediated allergic inflammation. Comprehensive reviews of the biology of IL-33 and its involvement in non-allergic conditions including autoimmune diseases and cancer have recently be described elsewhere (5, 9, 12).

## Regulatory mechanisms of IL-33 expression and its interaction with ST2

**Figure 1:**
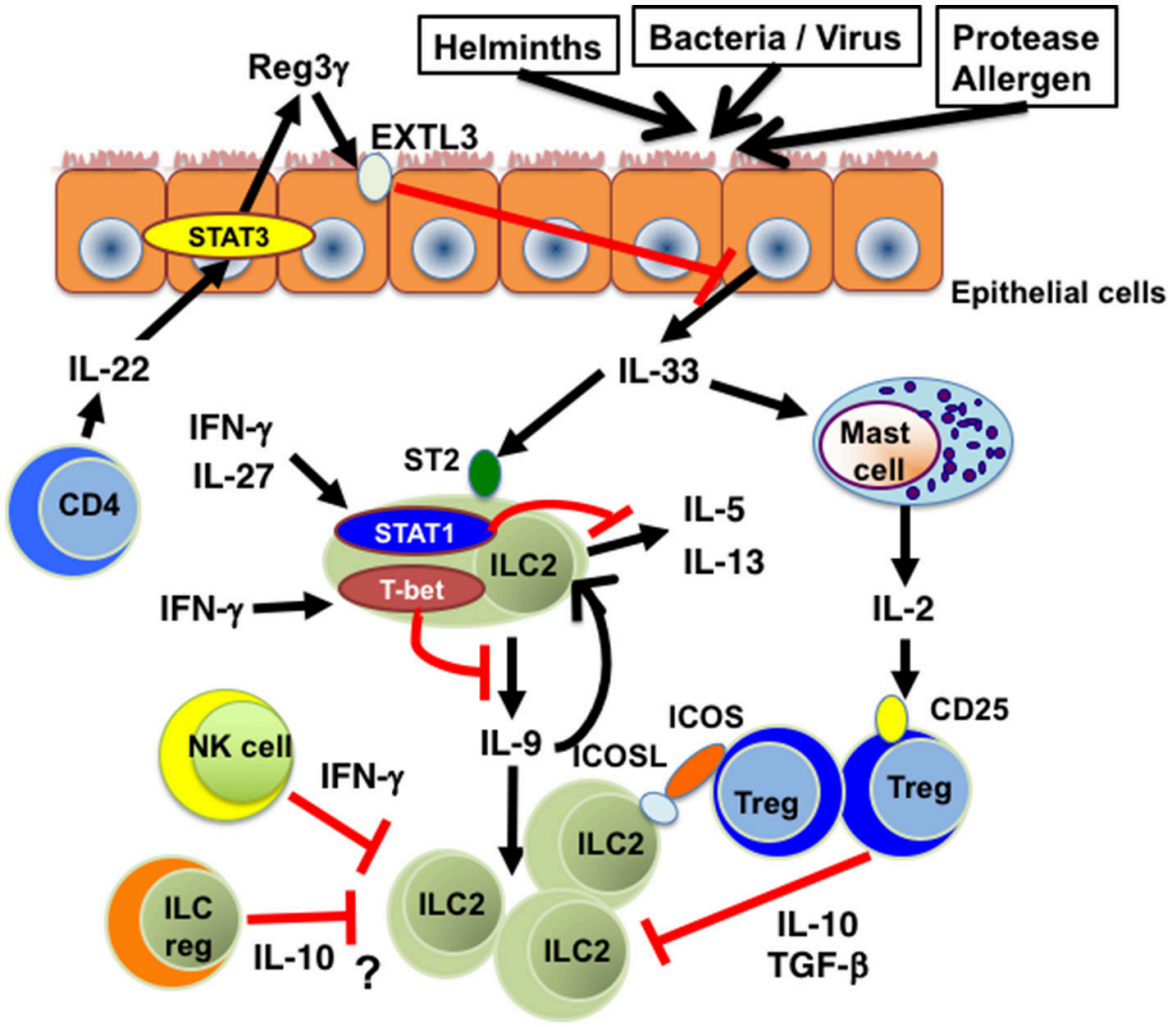
The inhibitory effects of cytokines on IL-33-ST2-ILC2 axis. Th1 cell-related cytokines such as IFN-γ and IL-27 inhibit IL-33-induced cytokine production and proliferation of ILC2s in a STAT1-dependent manner. T-bet expressed in ILC2s suppresses IL-9 production and subsequent expansion of ILC2s. IL-22 produced by CD4^+^ T cells suppresses IL-33 production from lung epithelial cells through Reg3γ induction. IL-2 produced by IL-33-stimulated mast cells promotes the expansion of Tregs, which suppress ILC2-mediated inflammation through the production of IL-10 and TGF-β. ICOS-ICOSL-mediated cell contact is also involved in Treg-mediated ILC2 suppression. In addition, IFN-γ produced by NK cells inhibits cytokine production and function of ILC2s. Moreover, IL-10-producing ILCregs could be a suppresser of IL-33-ST2-ILC2-mediated inflammation. EXTL3, exostatin-like 3; ICOS, inducible T-cell costimulator; ICOSL, inducible T-cell costimulator ligand; ILC2, group 2 innate lymphoid cells; ILCreg, regulatory innate lymphoid cell. NK cell, natural killer cell; Reg3γ, regenerating islet-derived protein 3 gamma; STAT1, signal transducer and activator or transcription 1; STAT3, signal transducer and activator or transcription 3; ST2, suppression of tumorigenicity 2; T-bet, T-box transcription factor expressed in T-cells; Treg, regulatory T cell.

Bioactivity of IL-33-ST2 pathways is regulated by several distinct mechanisms (Figure 1). It has been shown that many of the single nucleotide polymorphisms (SNPs) in the human IL-33 gene associated with asthma are located in the promoter and intron 1, both of which are important for gene expression (17). Imai et al. have shown that keratinocyte-specific over-expression of IL-33 results in the spontaneous development of an atopic dermatitis (AD)-like skin disease, along with the activation of ST2^+^ ILC2s (18). In addition, transgenic mice expressing IL-33 by a keratin 14 promoter spontaneously developed keratoconjunctivitis (19). These findings suggest that the expression level of IL-33 is critical for the induction of allergic inflammation and thus the suppression of IL-33 expression seems to be a good therapeutic strategy for the treatment of allergic diseases. In this regard, we have recently reported that IL-22, which belongs to the IL-10 family of cytokines, induces the expression of Reg3γ from lung epithelial cells in mice and that Reg3γ suppresses HDM-induced IL-33 expression and accumulation of ILC2s in the lung (20) (Figure 1). These results indicate that the IL-22-Reg3γ pathway is one of endogenous mechanisms that inhibit IL-33 expression. TGF-β is also shown to inhibit IL-33 expression in lung epithelial cells (21).

Recent studies indicate that microRNAs (miRNAs) also play pivotal roles in the regulation of the IL-33-ST2 axis. miRNAs are short noncoding RNAs that regulate the expression of various cytokines and pathogen recognition receptors at post-transcriptional levels by binding to the 3′-untranslated region (UTR) (22, 23). Regarding the regulation of IL-33 expression, Tang et al. have reported that miR-200b and miR-200c are significantly reduced in asthmatic patients and the induction of miR-200b and miR-200c decreases IL-33 expression in lung epithelial cells by binding 3′UTR of IL-33 mRNA (24). miR-487b has also been shown to suppress IL-33 expression in bone marrow-derived macrophages (25) and lung epithelial cells (26). On the other hand, RNA binding protein Mex-3B upregulates IL-33 expression by inhibiting miR-487b-3p-mediated repression of IL-33 (26). Additionally, it has been shown that miR-155^−/−^ mice exhibit impaired IL-33 levels in lung in response to allergen challenge, suggesting the involvement of miR-155 in the induction of IL-33 expression (27). These findings indicate that multiple miRNAs are involved in the regulation of IL-33 expression during allergic responses.

**Figure 2:**
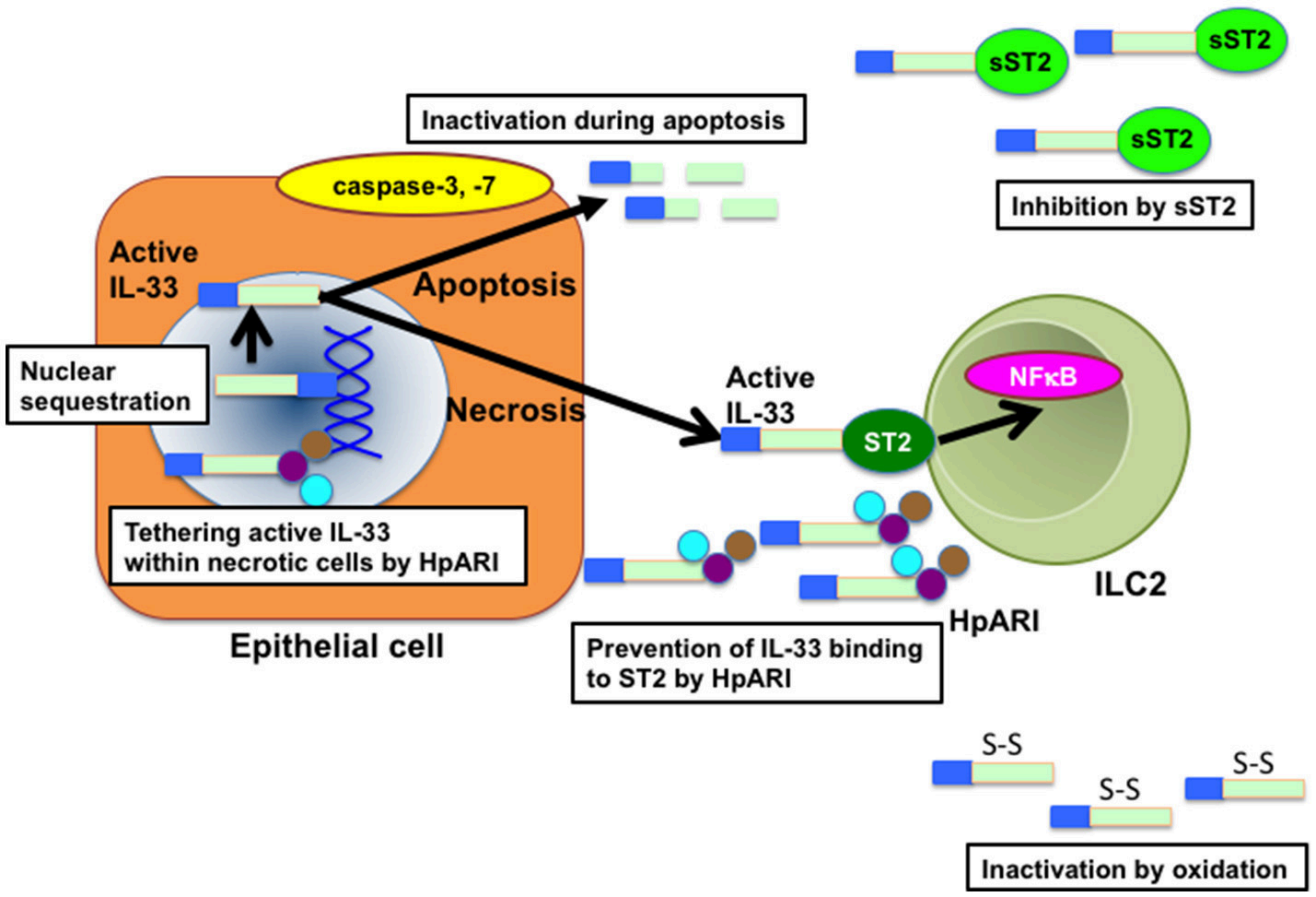
Molecular mechanisms that affect IL-33 bioactivity. Nuclear localization of IL-33 by binding to histone and chromatin is essential for the suppression of IL-33 bioactivity. At cell necrosis, active IL-33 is released from the nuclei of producing cells. Released active IL-33 is abrogated by several mechanisms such as inhibition by decoy receptor sST2 and inactivation by the conformational change. In addition, active IL-33 is cleaved and inactivated by caspases such as caspase-3 and caspase-7 during apoptosis of producing cells. HpARI secreted by *H. polygylus* inhibits the release of IL-33 by binding directly to active IL-33 and nuclear DNA, tethering active IL-33 within necrotic cells. HpARI also prevents binding of active IL-33 to ST2. HpARI, *Heligmosomooides polygyrus* alarmin release inhibitor; NFκB, nuclear factor-kappa B. sST2, soluble suppression of tumorigenicity 2.

The bioactivity of IL-33 is also regulated by posttranslational modification of IL-33 (5) (Figure 2). IL-33 is known to accumulate in the nucleus of producing cells by binding to histones and chromatin (1, 3, 28, 29). The N-terminal domain of IL-33 contains a chromatin-binding motif and a predicted nuclear localization sequence (28, 29). Importantly, the deletion of N-terminal domain containing a chromatin-binding motif has been shown to lead to early lethality in mice by constitutive secretion of IL-33 in serum, which causes severe multi-organ inflammation with infiltration of various immune cells including eosinophils, monocytes, neutrophils, and macrophages (30). These findings indicate that sequestration of IL-33 in the nucleus of IL-33-producing (or necrotic) cells is important to avoid the constitutive release of IL-33 and subsequent systemic inflammation. Interestingly, Osbourn et al. have recently shown that a product secreted by the parasite *Heligmosomoides polygyrus* (*H. polygyrus* Alarmin Release Inhibitor; HpARI) binds to IL-33 and also to nuclear DNA via its N-terminal complement control protein module pair (CCP1/2), tethering active IL-33 within necrotic cells, preventing its release, and inhibiting initiation of allergic responses (31). These findings suggest that *H. polygyrus* may escape from IL-33-mediated anti-parasitic responses via the production of HpARI.

It has also been reported that processing of IL-33 by proteases is crucial to regulate the bioactivity of IL-33 (Figure 2). IL-33 is cleaved by caspase-3 and caspase-7 during apoptosis, which generate two inactive IL-33 products (32–34). In addition, caspase-1 has been shown to cleave and inactivate IL-33 (32, 35, 36). Consistently, the administration of IL-33 greatly enhanced HDM-induced eosinophilic inflammation in caspase-1-deficient mice as compared with that in wild-type (WT) mice (36). Interestingly, cleaved chitin by acidic mammalian chitinase (AMCase) promotes the inactivation of IL-33 by inducing the activation of caspase-1 and subsequent activation of caspase-7, whereas uncleaved chitin promotes the release of IL-33 (35–37). Moreover, Cayrol et al. have recently shown that full-length IL-33 is cleaved by proteases derived from various environmental allergens such as fungi, HDM, cockroaches, and pollens and that the cleaved short mature forms of IL-33 are potent inducers of allergic airway inflammation (38). These findings suggest that the bioactivity of IL-33 is intricately regulated by endogenous and exogenous proteases.

The bioactivity of IL-33 is also controlled by the regulation of IL-33 binding to the receptor (Figure 2). IL-33 is shown to be rapidly inactivated in the extracellular lung environment by the oxidation of four critical cysteine residues and the formation of two disulfide bridges in the IL-1-like cytokine domain, resulting in an extensive conformational change that leads to the disruption of ST2 binding site (39). On the other hand, soluble spliced variant of ST2 (sST2), which directly binds to IL-33 and inhibits IL-33 bioactivity as a decoy receptor (40–42) is highly produced by mast cells and Th2 cells in asthmatic patients (40).

IL-33-ST2 signaling is also inhibited by SIGIRR (single immunoglobulin IL-1R-related molecule) which forms a complex with ST2 upon IL-33 stimulation (43). FBXL19, an orphan member of the Skp1-Cullin-F-box family of E3 ubiquitin ligases, selectively binds to ST2 to induce polyubiquitination and subsequent degradation of ST2 (44). Interestingly, IL-33 itself diminishes ST2 expression in mouse lung epithelial cells (MLE12 cells) in an FBXL19-dependent manner as one of negative feedback mechanisms (44).

## Regulatory mechanisms of IL-33-ST2-ILC2 axis

Recent studies have shown that IL-33-ST2-ILC2s axis is inhibited by several distinct mechanisms (Figure 1). It has been reported that pro-inflammatory cytokines such as type 1 interferons and Th1 cell-inducing cytokines such as IFN-γ and IL-27 inhibit IL-33-ST2-ILC2s axis *in vivo* as well as *in vitro*. Type 1 interferons including IFN-β suppress the proliferation and cytokine production of IL-33-activated murine and human ILC2s in a manner dependent on interferon-stimulated gene factor 3 (ISGF3) (45). Both IFN-γ and IL-27 have also been shown to inhibit cytokine production by ILC2s in IL-33-injected mice in a STAT1-dependent manner (45–48). In addition, STAT1-deficient mice have been shown to exhibit significantly increased IL-33 expression as compared with WT mice during respiratory syncytial virus (RSV) infection (49).

Both IFN-γ and IL-27 induce the expression of T-bet, a master regulator of Th1 cells (50), in CD4^+^ T cells in a STAT1-dependent manner (51, 52). In this regard, we found that T-bet is expressed in lung ILC2s upon IFN-γ stimulation and that T-bet inhibits IL-33-induced accumulation of ILC2s in the lung and subsequent eosinophilic airway inflammation (16). Importantly, IL-9 production is significantly enhanced in T-bet^−/−^ ILC2s and the neutralization of IL-9 markedly attenuates IL-33-induced eosinophilic airway inflammation in T-bet^−/−^ mice (16). These results indicate that T-bet expressed in ILC2s plays an inhibitory role in IL-33-ST2-induced accumulation of lung ILC2s by inhibiting IL-9 production, consistent with a previous report showing that IL-9 promotes cell survival of ILC2s in an autocrine mechanism (53).

Regarding the other extracellular factors involved in the regulation of IL-33 bioactivity, it has been shown that prostaglandin D2 (PGD2) in combination with IL-33 stimulates IL-13 secretion and ST2 expression in human ILC2s through CRTH2 (chemoattractant receptor-homologous molecule expressed on Th2 cells) (54, 55). On the other hand, lipoxin A4 (LXA4), which is a natural pro-resolving ligand for ALX/FPR2 receptors, decreases cytokine production by ILC2s in response to PGD2 and IL-33 (56). PGE2 and PGI2 have also been shown to exhibit a profound inhibitory effect on IL-33-mediated ILC2 expansion and cytokine production in mice and humans (57–59). Mechanistic studies revealed that PGE2- EP4 (E-prostanoid 4)-cyclic AMP signaling leads to the suppression of GATA3 and ST2 expression in ILC2s (59). These reports suggest that lipid mediators also seem to be critical factors for modulation of IL-33-ST2-ILC2 axis.

In addition, Laffont et al. have reported that androgen receptor-mediated signaling decreases the numbers of ILC2 progenitors in bone marrow and suppresses IL-33-induced ILC2-mediated airway inflammation (60). Moreover, upon IL-33 administration, the number of ILC2s is significantly increased in gonadectomized male mice as compared to sham-operated male mice (61). Consistently, testosterone attenuates alternaria extract-induced cytokine expression of ILC2s and eosinophils infiltration by decreasing the numbers of lung ILC2s (61). These results indicate that the imbalance in sexual hormones could be involved in the regulation of IL-33-mediated inflammatory diseases.

Recently, Moriyama et al. have reported that murine ILC2s express high levels of β2-adrenergic receptor (β2AR) (62) and the treatment with β2AR agonist (salmeterol) inhibits IL-33-induced IL-5 and IL-13 production of ILC2s, suggesting that the IL-33-ST2-ILC2 axis could be regulated by nervous systems including β2AR signaling (63). Moreover, Thio et al. have shown that butyrate inhibits IL-33-induced GATA3 expression and cytokine production in ILC2s (64). On the other hand, Suzuki et al. have shown that IL-33 enhances the expression of Spred1 (sprouty-related Ena/VASP homology 1 domain-containing protein) in ILC2s, which negatively regulates ILC2 expansion and cytokine production by suppressing the Ras-ERK pathway (65).

## Immune cell populations involved in the regulation of IL-33-ST2-ILC2 axis

Several cell types have been shown to regulate IL-33-ST2-ILC2 axis (Figure 1) in allergy. Roy et al. have shown that chymase derived from mast cells degrades IL-33 and that IL-33-induced TNF-α response is enhanced in chymase-deficient mice compared with WT mice (66). Moreover, it has been shown that IL-2 produced by IL-33-stimulated mast cells promotes the expansion of IL-10-producing CD4^+^ CD25^+^ Foxp3^+^ Tregs, thereby suppressing the development of IL-33-induced airway eosinophilia (67). These findings suggest that mast cells suppress IL-33-ST2-mediated inflammation through at least two distinct mechanisms. On the other hand, Rigas et al. have shown that iTregs attenuate IL-33-induced airway hyperreactivity by inhibiting IL-5 and IL-13 production from ILC2s and that the interaction of inducible T-cell costimulator ligand (ICOSL) on ILC2s with ICOS on Tregs is required for Treg-mediated suppression of ILC2 function (68). IFN-γ-producing NK cells also inhibit the cytokine expression and function of ILC2s (69).

More recently, Wang et al. have demonstrated that a regulatory subpopulation of ILCs (called ILCregs), which produce IL-10 and TGF-β, exists in murine and human intestine (70) and that ILCregs suppress intestinal inflammation driven by ILC1s and ILC3s via the secretion of IL-10 (70). Although it is reported that ILCregs isolated from the intestine do not suppress IL-33-induced IL-5 and IL-13 production from ILC2s (60), it remains unclear whether ILCregs are involved in the down-regulation of ILC2-mediated allergic responses *in vivo*.

## Potential clinical applications of IL-33-ST2 axis inhibition for allergic diseases

Regarding the IL-33-ST2 axis in human allergic diseases, genome-wide association studies have clarified that a variety of SNPs in IL33 gene and IL1RL1 (ST2) gene are associated with asthma susceptibility (17). Additionally, Savenije et al. have reported that SNPs of IL1RAP and TRAF6, a downstream molecule of the IL-33-ST2 pathway, are associated with a phenotype of intermediate-onset wheeze, which is closely associated with sensitization (71), suggesting that polymorphisms of genes involved in IL-33-ST2 axis are associated with onset of asthma in childhood. Consistently, it has been shown that serum sST2 levels correlate well with the severity of asthma exacerbation (72). Moreover, a rare loss of function mutation in IL-33 gene has been shown to cause reduced numbers of eosinophils in blood and to protect against asthma (73). These finding suggest that the IL-33-ST2 axis seems to be a rational target in the treatment of allergic diseases.

Based on these findings, biologics including three anti-IL-33 monoclonal antibodies (ANB020; AnaptysBio, AMG282; Genentech, and REGN3500; Regeneron Pharmaceuticals) and an anti-ST2 monoclonal antibody (GSK3772847; GlaxoSmithKline) have already been under development and clinical trials have commenced (http://www.clinicaltrials.gov/). A phase 2 study of ANB020 for peanut allergy have been completed, and subjects are currently being recruited in phase 2 trials for severe eosinophilic asthma and atopic dermatitis. Phase 1 trials of AMG282 for mild atopic asthma and chronic rhinosinusitis with nasal polyps have also been completed. In addition, REGN3500 is being examined for asthma in a phase 1 trial. ST2 -targeting therapies with GSK3772847 for asthma are also currently in clinical trials. Because it is currently difficult to delete ILC2s *in vivo*, the neutralization of IL-33-ST2 pathway is a practical approach to regulate IL-33-ST2-ILC2 axis for the treatment of refractory allergic diseases.

## Conclusion

In the past two decades, IL-33 has been regarded as an important initiator of immune responses. Additionally, recent studies have provided strong evidence that IL-33-ST2-ILC2s axis is one of central pathways in the pathogenesis of allergic inflammation. Accordingly, biologics targeting IL-33-ST2-ILC2 axis have already been under development. In addition, recent studies have shown that IL-33-ST2-ILC2s axis is negatively regulated by both cell intrinsic and extrinsic mechanisms. Given that IL-33-ST2-ILC2s axis seems to be deeply involved in the initiation phase of allergic diseases, these studies on the inhibitory mechanisms have yielded critical insights into the pathogenic mechanism underlying the onset of allergic diseases. Although further studies are needed to address how IL-33-ST2-ILC2s axis is negatively regulated in pathological situations in more detail, selective targeting of IL-33-ST2-ILC2s axis would be a promising strategy for the treatment of allergic diseases.

## Author contributions

All authors listed have made a substantial, direct and intellectual contribution to the work, and approved it for publication.

### Conflict of interest statement

The authors declare that the research was conducted in the absence of any commercial or financial relationships that could be construed as a potential conflict of interest.
